# Creative Arts-Based Therapies for Stroke Survivors: A Qualitative Systematic Review

**DOI:** 10.3389/fpsyg.2018.01646

**Published:** 2018-09-25

**Authors:** Temmy Lee Ting Lo, Janet Lok Chun Lee, Rainbow Tin Hung Ho

**Affiliations:** ^1^Department of Social Work and Social Administration, The University of Hong Kong, Hong Kong, Hong Kong; ^2^Centre on Behavioral Health, The University of Hong Kong, Hong Kong, Hong Kong; ^3^Sau Po Centre on Ageing, The University of Hong Kong, Hong Kong, Hong Kong

**Keywords:** creative arts-based therapies, expressive arts therapy, qualitative systematic review, rehabilitation, stroke

## Abstract

**Background:** Stroke is a life-threating cerebrovascular disease. Without proper and immediate treatment, it can cause long-term disabilities and even death. While current rehabilitation focuses on functional needs, it does not fully address the psychosocial issues. Creative arts-based therapies, however, may have the potential to be of assistance.

**Methods:** A systematic review was conducted to synthesize the qualitative findings of the stroke survivors' positive and negative experiences in participating in creative arts-based therapies. A systematic literature search was conducted across diverse databases. A thematic synthesis was adopted to analyze the results from different qualitative studies and mix-method studies.

**Results:** Among the 367 studies extracted from various databases, 11 studies met the inclusion criteria and were of acceptable quality. The following five analytical themes were identified: functional restoration, psychological support, social engagement, spiritual experience, and short-comings and barriers.

**Conclusion:** Creative arts-based therapies have demonstrated their strengths in addressing psychosocial needs for stroke survivors. Different art modalities are perceived to be useful in achieving different therapeutic goals. Therapies based on a single art modality or combined modalities have different specialties and characteristics. Further research is needed to demonstrate the differential benefits or special advantages of using single or multiple art modalities as well as having qualified therapists in creative arts-based therapies.

## Introduction

Stroke is a severe cerebrovascular disease. The World Health Organization (WHO) defined stroke as a disease that has “rapidly developing clinical signs of focal (or global) disturbance of cerebral function, with symptoms lasting 24 hours or longer or leading to death, with no apparent cause other than of vascular origin” (WHO MONICA Project Investigators, 1988). Without immediate and proper treatment, it can cause permanent physical disabilities such as paralyzed limbs, or even death. There are three main types of stroke, namely, ischemic stroke, hemorrhagic stroke, and transient ischemic attack (TIA). According to the American Heart Association (2018), ischemic stroke occurs when there is a blockage in the blood vessel which supplies blood to the brain. Hemorrhagic stroke refers to the fracture in a weakened blood vessel in the brain. Transient ischemic attack (TIA), also named “mini-stroke,” occurs when there is a temporary obstruction in the blood vessel(s) of the brain.

### Current situation of stroke

According to the latest global statistics, the prevalence rate of stroke was approximately 25.7 million and was the second leading cause of death in 2013 (Benjamin et al., 2017). Although older people have a higher risk of stroke, the onset of stroke among people aged 20–64 years has increased by 25% from 1990 to 2010 worldwide (Feigin et al., 2014). In the United States, around 795,000 people experience a new or recurrent stroke every year. Stroke is also the fifth leading cause of death and the leading cause of severe long-term disabilities in the United States (Benjamin et al., 2017). In Hong Kong, there are ~2 million patients with chronic illnesses and 2.2% of them are stroke patients (Census Statistics Department, 2015). Every year, ~3000 people die of stroke (Hospital Authority, 2018). Cerebrovascular diseases, including stroke, are the fourth leading causes of death in Hong Kong (Centre for Health Protection, 2018). These statistics indicate that the world is now facing a grave challenge with an increasing number of stroke survivors, thus more resources are needed for rehabilitation and post-stroke support.

### Current rehabilitation for stroke survivors

Current rehabilitation for stroke survivors depends heavily on physiotherapies, occupational therapies, and speech therapies. These rehabilitation programs are essential for stroke survivors as they aid in functional recovery. Physiotherapy, also recognized as physical therapy, focuses on the movement and physical function of patients and aims at the maintenance, development, and restoration of mobility (World Confederation for Physical Therapy, 2017). The main goal of occupational therapy is to facilitate patients to participate in their daily life activities and enhance their self-autonomy (World Federation of Occupational Therapy, 2012). Speech therapy aims at treating the patients' speech, language, and swallowing problems and at enhancing the patients' verbal communication and swallowing abilities (American Speech-Language-Hearing Association, 2018). Functional recovery is of utmost importance in stroke rehabilitation, especially during the first 6 months after the onset of stroke. The sudden loss of physical ability as well as the changes in various aspects of life caused by stroke also trigger psychological distress, social withdrawal and confusion in the meaning in life (Knapp et al., 2000; Yeung et al., 2011), which together need to be further addressed. These psychosocial and spiritual needs are also suggested to be crucial for the quality of life for stroke survivors (Katona et al., 2015). Enhancing the post-stroke quality of life has therefore been increasingly emphasized in recent years, in particular, in the United States (American Heart Association, 2013) and Australia (Australian Stroke Foundation, 2008). Due to their non-intrusive processes, therefore, non-pharmacological approaches, in particular, have been drawing considerable attention. Among them, creative arts-based therapies, which are mostly playful and without side-effects, have been recommended for stroke survivors. Different art modalities are also being suggested to be useful in stimulating different parts of the brain, and the stimulations from diverse art forms may further enhance the neuroplasticity in the brain and this may be helpful for facilitating the recovery process after stroke (Demarin, 2017). In addition, neurological evidence has shown that regularly listening to music after stroke may lead to structural changes in the brain among stroke survivors, and these structural changes may, in turn, relate to improvements in cognition (Särkämö et al., 2014). Moreover, engaging in a regular visual arts intervention has also been proven to facilitate the spatial improvement in functional connectivity in certain parts of the brain which may be associated with the psychological resilience in adults (Bolwerk et al., 2014). Apart from the above evidence, dance movement has also been suggested as an innovative approach for the rehabilitation for stroke survivors, due to its nature of engaging both physical and cognitive functions, dance may thus have the potential to tackle both the physical and cognitive impairments simultaneously (Dhami et al., 2015).

### Arts-based therapeutic approaches

There are various types of arts-based therapeutic approaches. While creative arts-based therapies emphasize specific art forms such as music therapy, art therapy, dance movement therapy, drama therapy, and expressive writing, other approaches such as expressive arts therapy make use of the integrative approach and multiple art forms. Although each approach has its own uniqueness and characteristics, all arts-based interventions have a common goal of providing stimulation of different sensations, cultivate a safe environment for self-exploration, and encourage self-expression, creativity, and imagination through the use of arts. Over the past decades, an increasing number of studies have been conducted in examining the outcomes of creative arts-based therapies for stroke survivors. Several systematic reviews and literature reviews on the outcomes of specific types of creative arts-based therapies for this population have also been published. For instance, Strzemecka (2013) completed a review of music therapy in stroke rehabilitation, focusing on the role of different types of music therapy in stroke rehabilitation. Reynolds (2012) also published a review of art therapy for stroke survivors, which gathered both quantitative and qualitative findings from various studies. Nevertheless, these review articles only focused on a single art modality approach. In this qualitative systematic review, however, different forms of creative arts-based therapies were included, as there appears to be a paucity of transparent understanding of the beneficial outcomes of different creative arts-based therapies. With a better understanding of the characteristics and specialties of therapies using different art modalities, art therapists, service providers, and users, may afford a better appreciation on how creative arts-based therapies can provide the most favorable experience to stroke survivors.

The present qualitative systematic review serves the specific aim of examining both the positive and negative experiences of creative arts-based therapies for stroke survivors. As the stroke survivors' personal and unique experiences of participation in creative arts-based therapies can provide in-depth information on their perspectives, the present review has thus adopted the qualitative approach.

## Methods

This qualitative systematic review was conducted based on the framework in the Preferred Reporting Items for Systematic Reviews and Meta-analyses Protocols (“PRISMA-P”) (Moher et al., 2015).

### Definition of key words

Creative arts-based therapies refer to interventions that usually apply one major art form as a medium to achieve a physiological, psychosocial, social, or any other therapeutic goals. Hence, the type of art form can include music, dance movement, visual arts, creative writing, and drama. Creative arts-based therapies designed for survivors of the two major strokes, namely, ischemic stroke, and hemorrhage stroke, were included in the review because the severity and effects of the TIAs are short-lived.

### Search strategy

A literature review of publications on creative arts-based therapies for stroke survivors was conducted. This qualitative review was based on the materials retrieved from a systematic literature search using six computerized databases, namely, PsycINFO, PubMed, MEDLINE, CINAHL, Cochrane, and Embase. The search was completed on February 20, 2018 and there were no restrictions on the publication period. The search was based on whether the combination of the following keywords appeared in any abstract: “stroke” and “music OR art OR dance OR dance movement OR drama OR writing” and “qualitative OR interview OR focus group OR observation OR thematic analysis OR grounded theory OR content analysis OR framework approach OR phenomenographic.” The key words for selecting the materials related to any kind of creative arts-based therapies were inspired by Beard (2012), who conducted a systematic review in dementia care that also required different kinds of creative arts-based therapies. To enhance the coverage and accuracy of the studies, reference lists of the included studies were also screened to extract relevant articles that were overlooked in the databases. Manual searches on the internet and the Google Scholar website were also performed.

### Eligibility criteria

Studies included in this review were based on the inclusion criteria listed in Table 1.

**Table 1:** Inclusion criteria of reviewed articles.

**Criteria**	**Description**
Study types	Qualitative studies, mixed-method studies, and quantitative studies supplemented with qualitative results were included. Studies without the full text, abstracts presented in the conferences, brief reports, and unpublished theses were excluded in this review
Study designs	There were no restrictions on the study designs, and studies with any qualitative elements intended to record the stroke survivors' perceived experiences or perspectives in participating in creative-arts based therapies, were included.
Participants	Patients diagnosed with two major types of strokes: hemorrhage stroke or ischemic stroke. No restrictions on age and nationality were imposed.
Creative arts-based therapies	Any intervention that applied one art modality as a medium to achieve any therapeutic goal. The interventions could be delivered in a group or individual formats.
Interventionists	Interventions both guided by qualified creative arts therapists and by other professionals were included.
Duration	There were no limitations on the length or duration of the interventions.
Setting	There were no restrictions on the setting of the interventions. They could be conducted in any venue such as a hospital, or anywhere within the community setting.
Language	Only articles written in English were included in this review.

### Data extraction

The process of data extraction began with comprehensive searches on different databases and from other resources. All searches were run independently in each database. Each result from each database was extracted into EndNote. For the first screening, duplicated studies were identified and removed. For the second screening, titles, and abstracts of the studies were examined. Studies, however, were excluded if the titles were not relevant. For the third screening, full texts of the remaining studies were examined by the first two authors, LTLT and LJLC, based on the eligible criteria. Appropriate reasons were also provided for any excluded studies. The last screening was the quality assessment and was conducted by LTLT in cooperation with LJLC. Data extractions were conducted on the final remaining studies. In order to enhance accuracy and to avoid any bias, LTLT and LJLC also jointly conducted the data extraction process. Data extractions were based on a tailor-made Excel file that included the study design, data collection methods, setting, informants, interventionists, use of art forms, samples, and sampling methods.

### Data synthesis

The data synthesis process adopted the thematic synthesis approach developed by Thomas and Harden (2008) which involves three steps: the first step is to conduct line-by-line coding, the second, to develop descriptive themes, and the last, to generate analytical themes. The result sections from the included studies were then extracted for free line-by-line coding. The two authors, LTLT and LJLC, conducted the coding independently. The two authors then joined together to discuss the consistencies and discrepancies of the codes and the interpretation of the findings. Subsequently, the two authors decided on the descriptive themes based on the similarities and differences of the preliminary codes. Finally, after several rounds of discussion, the analytic themes were developed. A summary of the results based on the decided descriptive and analytical themes was then drafted by LTLT and reviewed by LJLC and HRTH together. All discrepancies throughout the data synthesis process were resolved by consensus. Quotations from the studies were also extracted to support the descriptive and analytical themes.

## Results

### Selection flow

**Figure 1:**
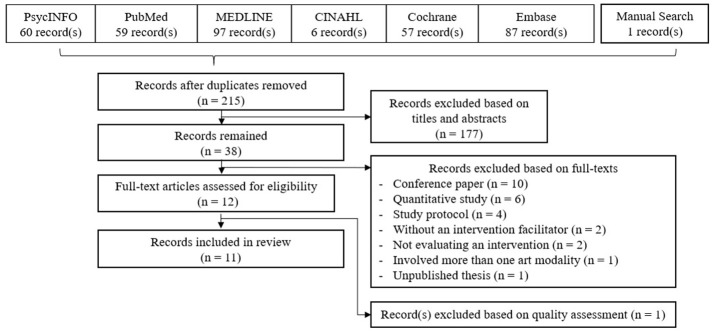
PRISMA flow diagram.

After searching for relevant materials, 367 studies were identified. After eliminating duplicated studies, 215 studies were selected. After screening through title and abstract, 38 studies were selected, and their full text were examined. After that, 26 studies were further excluded with reasons provided. The remaining 12 studies underwent quality assessment, after which one study was excluded (Demers and McKinley, 2015). Finally, 11 studies were identified and included in the qualitative systematic review. The process of identification and selection of inclusion materials is illustrated in Figure 1. The flow diagram is based on the PRISMA guideline.

### Quality assessments

To evaluate the quality of qualitative research suggested by Murphy et al. (1998), the quality of the retrieved materials was based on two main aspects, namely, credibility and relevance. The credibility of the studies was assessed by the following five criteria: appropriate sampling and data collection method, auditability, reflexivity, handling negative cases, and fair dealing (Murphy et al., 1998). The relevance of the studies was assessed by the following two criteria: transferability and analytic generalization (Murphy et al., 1998). This quality assessment method was adopted from a qualitative meta-synthesis study on the life experiences of stroke survivors conducted by Salter et al. (2008). Thus, studies which did not meet the standards of the two main aspects, namely, credibility or relevance, were excluded. Quality assessments were also conducted by LTLT and LJLC independently. Results of the quality assessments were later discussed among themselves and their disagreements resolved by consensus. Supplementary Material (Table S1) presents the results of the quality assessments.

### General characteristics

Eleven studies published between 2005 and 2017 were included in the systematic review. Table 2 lists the general characteristics of those studies. Eight studies were qualitative studies and three were mixed-method studies. The studies were conducted in different countries and cities which included Australia, Brazil, Hong Kong, New Zealand, Sweden, the United Kingdom, and the United States. Nine studies were conducted in a community setting and two in a hospital setting. Six studies applied the convenience sampling method, three adopted the purposive sampling method, and two used the voluntary sampling method for sample recruitment. Nine studies involved semi-structured interviews for data collection, three used focus group interviews, two involved observations, and one applied structured interviews.

**Table 2:** Descriptive information of included studies (*N* = 11).

		***f***	**Corresponding studies**
Research types	Qualitative	8	Higgins et al., 2005; Beesley et al., 2011; Guerrero et al., 2014; Sit et al., 2014; Thornberg et al., 2014; Morris et al., 2016; Tarrant et al., 2016; Wolff et al., 2017
	Mixed-method	3	Tamplin et al., 2013; Fogg-Rogers et al., 2016; Street et al., 2017
Countries/Cities	United Kingdom	4	Higgins et al., 2005; Morris et al., 2016; Tarrant et al., 2016; Street et al., 2017
	Australia	2	Beesley et al., 2011; Tamplin et al., 2013
	Brazil	1	Wolff et al., 2017
	Hong Kong	1	Sit et al., 2014
	New Zealand	1	Fogg-Rogers et al., 2016
	Sweden	1	Thornberg et al., 2014
	United States	1	Guerrero et al., 2014
Setting	Community	9	Beesley et al., 2011; Tamplin et al., 2013; Guerrero et al., 2014; Sit et al., 2014; Thornberg et al., 2014; Fogg-Rogers et al., 2016; Tarrant et al., 2016; Street et al., 2017; Wolff et al., 2017
	Hospital	2	Higgins et al., 2005; Morris et al., 2016
Sampling methods	Convenience sampling	6	Beesley et al., 2011; Tamplin et al., 2013; Guerrero et al., 2014; Fogg-Rogers et al., 2016; Tarrant et al., 2016; Street et al., 2017
	Purposive sampling	3	Higgins et al., 2005; Sit et al., 2014; Morris et al., 2016
	Voluntary sampling	2	Thornberg et al., 2014; Wolff et al., 2017
Data collection	Semi-structured interviews	9	Higgins et al., 2005; Beesley et al., 2011; Tamplin et al., 2013; Sit et al., 2014; Thornberg et al., 2014; Fogg-Rogers et al., 2016; Morris et al., 2016; Tarrant et al., 2016; Wolff et al., 2017
	Focus group interviews	3	Beesley et al., 2011; Guerrero et al., 2014; Tarrant et al., 2016
	Observations	2	Higgins et al., 2005; Guerrero et al., 2014
	Structured interviews	1	Street et al., 2017

As to the details of the interventions, six studies applied music-based interventions, three applied visual arts-based intervention, one applied dance-based intervention, and one study applied literature-based intervention. Among the six music-based intervention studies, five music-based interventions were led by qualified music therapists, and one music-based intervention was led by music facilitators. Other art modalities interventions were led by artists, fine arts students, nurses, actors or actresses, and dance instructors. Other details of the interventions are listed in Table 3.

**Table 3:** Interventions' characteristics of included studies.

**Studies**	**Art forms**	**Interventionists**	**Interventions**			
			**Contents**	**Duration**	**Hours**	**Frequencies**
Beesley et al., 2011	Visual arts	Fine arts graduates; members of the community stroke team; assistants	Art groups	8 weeks	2 h	Weekly
Fogg-Rogers et al., 2016	Music	Music therapists; volunteers	Choral singing practices	6 months to 2 years	/	/
Guerrero et al., 2014	Music	Nordoff-Robbins music therapists; occupational therapists	Integrated music therapies and occupational therapies	6 weeks	45 min	Twice a week
Higgins et al., 2005	Literature	Professional actors	Individual, group reading sessions	/	Individual/ group: on average 20–21 min	/
Morris et al., 2016	Visual arts	Artists	Tayside Creative Engagement Intervention (“TCEI”)	8 weeks	Individual: 40 min; Group: 1.5 h	Weekly
Sit et al., 2014	Visual arts	Nurses	Leisure Art-based Creative Engagement (“LACE”)	7 weeks	2.5 h	Weekly
Street et al., 2017	Music	Neurologic music therapists	Therapeutic instrumental music performance (“TIMP”)	6 weeks	20–30 min	Twice a week
Tamplin et al., 2013	Music	Music therapists	Singing rehearsals	20 weeks	2 h with 30 min break	Weekly
Tarrant et al., 2016	Music	Music facilitators	Singing sessions	/	1.5 h	/
Thornberg et al., 2014	Music	Music therapists	Ronnie Gardiner Rhythm and Music Therapy (“RGRM”)	10 weeks	/	Weekly
Wolff et al., 2017	Dance	Dance instructors	Dance lessons	3 years	1 h	Weekly

“*/” represents unclear or not mentioned*.

### Themes

Overall, five analytical themes were identified, namely, “functional restoration,” “psychological support,” “social engagement,” “spiritual experience,” and “short-comings and barriers.” Each analytical theme was supported by its corresponding descriptive themes which are listed in Tables 4–8. Supporting quotations from stroke survivors, significant others, and interventionists were also retrieved to support each descriptive and analytical theme.

**Table 4:** Descriptive themes under “functional restoration.”

**Descriptive themes**	**Supporting studies**	**Selected supporting quotations**
- Improvement in physical abilities	Guerrero et al., 2014; Thornberg et al., 2014; Fogg-Rogers et al., 2016; Morris et al., 2016; Street et al., 2017; Wolff et al., 2017	Music - *[SS]* “*I can put my arm in a position which is easier for me to get dressed.” “I feel like my fingers have become more active, especially my thumb, the opening and closing of my thumb has definitely improved” (Street et al., 2017) (Q)* Visual arts - *[I] “I think when they are involved in doing something like painting, they are so involved in the creative process that they don't realize that maybe they are doing physical things, using their arms and thinking as well. I guess doing art helps their skills, with using their limbs, their hands and their sight, but they don't really think of it in that sense” (Morris et al., 2016) (NQ)* Dance - *[SS] “Leg, arm, foot […] and better speaking […] the exercises helped me a lot. […] sweeping, dusting, fixing the bed”(Wolff et al., 2017) (NQ)*
- Improvement in communication	Higgins et al., 2005; Tamplin et al., 2013; Fogg-Rogers et al., 2016; Morris et al., 2016; Wolff et al., 2017	Music - *[SO] “I think his speech is a little bit better than what it was. I mean he was always good, but he seems to have got clearer in his speech” (Tamplin et al., 2013) (Q)* Visual arts - *[SS] “One of the nurses says, before you came here, you couldn't say a word…you came in here and I asked you questions and you just pointed at things. But, however, after doing art you came out talking” (Morris et al., 2016) (NQ)*

*[SS] Stroke survivor; [SO] Significant other; [I] Interventionist; (Q) Intervention led by qualified therapist; (NQ) Intervention led by other professionals*.

**Table 5:** Descriptive themes under “psychological support.”

**Descriptive themes**	**Supporting studies**	**Selected supporting quotations**
- Self-expression	Higgins et al., 2005; Beesley et al., 2011; Guerrero et al., 2014; Sit et al., 2014; Morris et al., 2016	Visual arts - *[SS]“I enjoyed it [group project], I did find it challenging because you put so much of yourself into it. Yeah” (Beesley et al., 2011) (NQ)* Literature - *[SS] “I can cry at those stories and they're not even real, yet I can't cry at my own situation. But in a strange way I am glad, and I feel better afterwards” (Higgins et al., 2005) (NQ)*
- Enhancement in confidence	Beesley et al., 2011; Tamplin et al., 2013; Sit et al., 2014; Fogg-Rogers et al., 2016; Morris et al., 2016	Music - *[SS] “(The choir helps me) to regain my confidence to face people again” (Tamplin et al., 2013) (Q)* Visual arts - *[SS] “I realized I had underestimated my ability. I always thought I could no longer perform many things. But, it was not the case. The stroke may have affected some aspects of my ability. Hence, I am not doing as efficiently as before. However, this does not mean that I cannot perform as well as before. I am proud of my artwork and of myself” (Sit et al., 2014) (NQ)* - *[I] “People often say “Oh that's brilliant, that's amazing, I didn't know you could do that” and that again increases their self-esteem because they hear that coming from the artist or the staff and not from their own family, so that is a great confidence booster” (Morris et al., 2016) (NQ)*
- Enhancement in mood	Beesley et al., 2011; Tamplin et al., 2013; Guerrero et al., 2014; Sit et al., 2014; Fogg-Rogers et al., 2016; Morris et al., 2016; Street et al., 2017; Wolff et al., 2017	Music - *[SO] “They're all so happy when they are here. Nigel is basically a very placid person, so in that respect I'm very fortunate. He just seems to be so happy to go, when I usually say “yes we are going to the choir” he is happy (Tamplin et al., 2013) (Q)* Visual arts - *[SS] “It is not simply having fun. The way I concentrated in doing the artwork, putting thoughts into action, and creating and visualizing my own art piece gave me an opportunity to experience a deep sense of enjoyment that I have missed for a long time since the stroke” (Sit et al., 2014) (NQ)* Dance - *[SS] “Of course…yes it is…for the better, right? […] I feel happier” (Wolff et al., 2017) (NQ)*
- Relaxation and distraction	Higgins et al., 2005; Beesley et al., 2011; Morris et al., 2016; Tarrant et al., 2016	Visual arts - *[SS] “It was good to go there and relax and forget all about it [the struggles]” (Beesley et al., 2011) (NQ)* Literature - *[SS] “There's nothing to do except sit there…this panicky feeling would well up and I couldn't believe it was me there. So it was a relief to have the story sessions 'cos you would just forget for a bit and get caught up with the storyline” (Higgins et al., 2005) (NQ)*
- Encouragement	Higgins et al., 2005; Beesley et al., 2011; Morris et al., 2016; Street et al., 2017	Music - *[SS] “Very encouraging, good, satisfying” (Street et al., 2017) (Q)* Visual arts - *[SS] “It's helpful to see what other people deal with…because sometimes you feel like you are finished, but you're not” (Beesley et al., 2011) (NQ)*
- Connection with oneself	Higgins et al., 2005; Guerrero et al., 2014; Sit et al., 2014; Thornberg et al., 2014	Music - [SS] “*I've gained a feeling in my body” (Thornberg et al., 2014) (Q)* Visual arts - *[SS]* “*I can visualize the two sides of my body reconnecting in my drawing. After the stroke, this was the first time that I could sense the wholeness of my body” (Sit et al., 2014) (NQ)* Literature - *[SS] “I thought my life was over, you know. I would be somebody else now when I met folks. But I did feel like myself, I'm still from Lambeth, same as Mary (another patient)…I remembered all the same shops (mentioned in the story) as she did. It made me think well I'm still here somewhere after all!” (Higgins et al., 2005) (NQ)*
- Sense of control	Higgins et al., 2005; Thornberg et al., 2014; Morris et al., 2016	Music - *[SS] “I couldn't sit straight at all to start with, I had terrible vertigo all the time and because of that the…therapy was perfect” (Thornberg et al., 2014) (Q)* Visual arts - *[SS] “I was really surprised that I was able to do it so well, especially with my left hand. Because it took a while just to learn the right way to hold a pencil. Well my first thought was, thank God I could do something with my left hand, because I'm not left handed, I'm right handed” (Morris et al., 2016) (NQ)*

*[SS] Stroke survivor; [SO] Significant other; [I] Interventionist; (Q) Intervention led by qualified therapist; (NQ) Intervention led by other professionals*.

**Table 6:** Descriptive themes under “social engagement.”

**Descriptive themes**	**Supporting studies**	**Selected supporting quotations**
- Peer support	Beesley et al., 2011; Tamplin et al., 2013; Guerrero et al., 2014; Sit et al., 2014; Tarrant et al., 2016	Music - *[SS] “It was comforting and encouraging to be with people who are going through the same thing…and to be working on some-thing together” (Guerrero et al., 2014) (Q)* Visual arts - *[SS] “I sat next to Mary. Her left hand was disabled and mine's on the right so we could help each other. I hold the file pen with my left hand and she opened its cap with her right hand. We are good working partners. They (other participants) said we are twins” (Sit et al., 2014) (NQ)*
- Social interactions	Higgins et al., 2005; Beesley et al., 2011; Tamplin et al., 2013; Morris et al., 2016; Tarrant et al., 2016;	Visual arts - *[SS] “I was at a stage where I didn't want to go out anywhere…Because of all the obstacles of a disabled person, I just wasn't adapting very well…I just stayed at home and didn't go anywhere, so this group has allowed me to find the courage to get out and socialize, yeah” (Beesley et al., 2011) (NQ)*
•[-] Connection with society	Beesley et al., 2011; Tamplin et al., 2013; Fogg-Rogers et al., 2016; Wolff et al., 2017	Music - *[SO] “Anything that takes them [people with stroke] out of their comfort zone is a really good thing. Anything, like the choir. I can just think of so many people who should've been going to that choir, but they probably wouldn't leave the house” (Fogg-Rogers et al., 2016) (Q)* Dance - *[SS] “Yeah, birthday, weekend barbeque [.] And when I go out I talk a lot on the streets, I even talk too much…” (Wolff et al., 2017) (NQ)*

*[SS] Stroke survivor; [SO] Significant other; [I] Interventionist; (Q) Intervention led by qualified therapist; (NQ) Intervention led by other professionals*.

**Table 7:** Descriptive themes under “spiritual experience.”

**Descriptive themes**	**Supporting studies**	**Selected supporting quotations**
- Hope infusion	Beesley et al., 2011; Sit et al., 2014; Morris et al., 2016	Visual arts - *[SS] “Yeah…it does, sort of inspire me at home. Unfortunately for me I see myself at a level that I was at when I was in uni…I'm not even close anymore. So it [the art group] has given me a sort of a track to try and get there…It has opened that door for me to go on” (Beesley et al., 2011) (NQ)* •[-] *[SS]* “*It makes you feel good talking, makes you realize you're not alone in this and there is still hope for something better”* (Morris et al., 2016) *(NQ)*
- Maintain religious practices	Higgins et al., 2005	No quotations

*[SS] Stroke survivor; [SO] Significant other; [I] Interventionist; (Q) Intervention led by qualified therapist; (NQ) Intervention led by other professionals*.

**Table 8:** Descriptive themes under “short-comings and barriers.”

**Descriptive themes**	**Supporting studies**	**Selected supporting quotations**
- Demanding process	Beesley et al., 2011	Music - *[SS] “…terribly confusing but that is really what it is about…you must try no matter how it works” (Thornberg et al., 2014) (Q)* Visual arts - *[SS] “I didn't get the concept of what it was about…it was the fact that I thought I hadn't grasped the concept, so it was more ‘mind ability' to grasp things' (Beesley et al., 2011) (NQ)*
- Struggled to use affected limbs	Beesley et al., 2011	Visual arts - *[SS] “You know I mean I didn't use my right hand much during the course that I just did but I did use my right hand a little bit sometimes…I am trying to use it a little bit” (Beesley et al., 2011) (NQ)*
- Triggered sad memories	Higgins et al., 2005	No quotations
- Linked with disabilities	(Higgins et al., 2005)	Literature - *[SS] “It's bad enough being stuck like this without being made to feel like an idiot as well. I can read you know” (Higgins et al., 2005) (NQ)*
- Could not - genuinely accept appreciation	Morris et al., 2016	Visual arts - *[SS] “Well, they were kind, but everyone could see that it was a failure. I was really ashamed. Ashamed of my low level of achievement but also of my low persistence rate” (Morris et al., 2016) (NQ)*

*[SS] Stroke survivor; [SO] Significant other; [I] Interventionist; (Q) Intervention led by qualified therapist; (NQ) Intervention led by other professionals*.

#### Functional restoration

The theme “functional restoration” involves the following descriptive themes: “improvement in physical abilities” and “improvement in communication.” The informants stated that the process of creating arts provided opportunities for them to use their affected limbs (Beesley et al., 2011; Morris et al., 2016). Playing musical instruments was useful in physical rehabilitation, particularly with respect to fine movements such as finger movements (Guerrero et al., 2014; Street et al., 2017). Whereas dance and rhythmic movements guided by music facilitate the whole-body connection and coordination (Thornberg et al., 2014; Wolff et al., 2017). “Improvement in communication” was a common theme for studies that used singing intervention (Tamplin et al., 2013; Fogg-Rogers et al., 2016). Group setting in a visual arts-based intervention also enhanced communication ability (Morris et al., 2016).

#### Psychological support

The theme “psychological support” includes the following descriptive themes: “self-expression,” “enhancement in confidence,” “enhancement in mood,” “relaxation and distraction,” “encouragement,” “connection with oneself,” and “sense of control.” The informants felt that various art-based interventions such as music, visual arts, and literature were able to provide opportunities for self-expression (Higgins et al., 2005; Beesley et al., 2011; Guerrero et al., 2014; Sit et al., 2014; Morris et al., 2016). Different creative arts-based therapies enhanced stroke survivors' confidence in different ways. For instance, they regained their self-confidence in communicating with others through singing intervention (Tamplin et al., 2013; Fogg-Rogers et al., 2016), increased their self-esteem by creating their own artwork and receiving positive feedback from others (Beesley et al., 2011; Morris et al., 2016), and by improving their mobility status (Guerrero et al., 2014). Their enjoyable experiences while engaging in different art forms were also frequently mentioned (Beesley et al., 2011; Tamplin et al., 2013; Guerrero et al., 2014; Sit et al., 2014; Fogg-Rogers et al., 2016; Morris et al., 2016; Street et al., 2017; Wolff et al., 2017). Participating in various art modalities activities were thus helpful in enabling relaxation and provide distraction in a tense rehabilitation environment (Higgins et al., 2005; Beesley et al., 2011; Morris et al., 2016; Tarrant et al., 2016). The informants also sensed the encouragement to move forward with different art modalities (Higgins et al., 2005; Beesley et al., 2011; Morris et al., 2016; Street et al., 2017). Different art forms stimulated different sensations, reconnecting the informants with their inner selves and their affected body parts (Higgins et al., 2005; Guerrero et al., 2014; Sit et al., 2014; Thornberg et al., 2014). The informants also mentioned regaining a sense of control that came from the opportunity to exercise a little control over the reading intervention (Higgins et al., 2005) and noticing an improvement in mobility during visual arts (Morris et al., 2016) or music-based intervention (Thornberg et al., 2014).

#### Social engagement

The theme “social engagement” is comprised of the following descriptive themes, “peer support,” “social interactions,” and “connection with society.” The informants expressed that the homogeneity of the group cultivated a common understanding, which in turn, enhanced peer support (Beesley et al., 2011; Tamplin et al., 2013; Guerrero et al., 2014; Sit et al., 2014; Tarrant et al., 2016) and provided an appropriate and safe occasion for social interactions (Higgins et al., 2005; Beesley et al., 2011; Tamplin et al., 2013; Morris et al., 2016; Tarrant et al., 2016). The informants further elaborated that individual sessions facilitated communication with interventionists and that a rapport was built so that they could talk freely about their concerns with their interventionists (Higgins et al., 2005). The informants also mentioned that they were not only encouraged to interact within the intervention sessions but were also encouraged to leave their homes regularly, reconnect with society, and resume normal social activities (Beesley et al., 2011; Tamplin et al., 2013; Fogg-Rogers et al., 2016; Wolff et al., 2017).

#### Spiritual experience

The theme “spiritual experience” involves the following descriptive themes: “hope infusion” and “maintain religious practices.” The informants described that the process of creating artwork inspired them to believe that they still had hope in the world (Beesley et al., 2011; Sit et al., 2014; Morris et al., 2016). Reading religious materials further encouraged informants to restore a certain level of religious practices (Higgins et al., 2005).

#### Short-comings and barriers

Although the effect of art-based interventions seems promising, the process of creating art and appreciating the artwork of others may also bring about negative effects. The findings of the studies also captured themes on the barriers encountered. The themes included “demanding process,” “struggled to use affected limbs,” “triggered sad memories,” “linked with disabilities,” and “could not genuinely accept appreciation.” One of the key barriers was that the process of creating and engaging using art may be too cognitively demanding (Beesley et al., 2011; Thornberg et al., 2014). The struggle to use their affected limbs could also be challenging and frustrating (Beesley et al., 2011). Reading through literature also sometimes brought back sad memories from the past (Higgins et al., 2005). Some informants also reported that they felt embarrassed when others praised their artwork as they themselves believed that it was terrible (Morris et al., 2016).

## Discussion

This review systematically reviewed qualitative studies and mixed-method studies of creative arts-based therapies with stroke survivors. Based on the literature search, 11 studies matched the inclusion criteria and were reviewed in detail. The major objective of this review was to organize the positive and negative experiences the stroke survivors had when they joined the creative arts-based therapies.

Regarding positive experiences, the reviewed studies provided promising findings that creative arts-based therapies brought about a certain degree of physical, psychological, social, and spiritual benefits. These interventions demonstrated their specialties and strengths in taking care of the stroke survivors' psychological issues such as boosting their self-confidence, providing motivation, and creating enjoyment for them. Furthermore, interventionists and stroke survivors' significant others also reported similar benefits from their observations and perspectives. Thus, service providers may consider using creative arts-based therapies in conjunction with existing rehabilitation programs for stroke survivors to help them recover not only physically but also psychologically and spiritually.

Regarding the types of arts used in the reviewed studies, around half of the studies applied music-based interventions (Tamplin et al., 2013; Guerrero et al., 2014; Thornberg et al., 2014; Fogg-Rogers et al., 2016; Tarrant et al., 2016; Street et al., 2017). Other studies applied visual arts (Beesley et al., 2011; Sit et al., 2014; Morris et al., 2016), dance (Wolff et al., 2017), and reading (Higgins et al., 2005). No studies were found to apply drama-based intervention though. Furthermore, each art form appears to have its own strengths and uniqueness in bringing about certain types of perceived benefits and experiences of the stroke survivors. For example, music and singing were reportedly very effective in helping stroke survivors regain their communication abilities (Tamplin et al., 2013; Fogg-Rogers et al., 2016); rhythmic movement guided by music and dance activities worked well for training mobility for the whole body (Thornberg et al., 2014; Wolff et al., 2017); playing musical instruments (Guerrero et al., 2014; Street et al., 2017) and creating artwork (Beesley et al., 2011; Morris et al., 2016) were able to challenge stroke survivors to use their affected limbs. Besides, visual arts-based interventions were perceived as more likely to infuse hope and faith among stroke survivors (Sit et al., 2014; Morris et al., 2016). Thus, this evidence suggested that different forms of creative arts-based interventions would be able to bring about different positive experiences on stroke survivors. Future arts-related interventions for stroke survivors may include single art form or a combination of different art modalities based on the needs and expectations of the stroke survivors.

Over the past decade, expressive arts-based therapy that uses multiple art modalities during the intervention process has been receiving increasing attention. Application of different art forms offers greater freedom and flexibility. The therapists can use different art modalities based on the participants' reactions, group dynamics, topics, and themes. Baumann et al. (2013) conducted a study to evaluate the effectiveness of a person-centered arts program for hospital-based stroke survivors. Their intervention involved visual arts, literature, music, and dance and movement. The stroke survivors appreciated the personalization of the intervention. It also helped cultivate the meaning and articulation between the art processes and themselves, which may further facilitate the development of new perspectives and insights. Kongkasuwan et al. (2016) conducted a randomized controlled trial to evaluate the effectiveness of creative arts therapy on more than 100 stroke survivors. The therapeutic program also involved diverse art modalities such as music and visual arts. This study showed that survivors participating in creative arts therapy in addition to conventional physiotherapy experienced lower depression levels, an enhanced quality of life, and an improvement in physical functions compared with survivors who had participated in physiotherapy alone.

Regarding short-comings and barriers, the stroke survivors felt that the experiences could sometimes be demanding and challenging. Similar to conventional physiotherapies, arts-based interventions can also cause physical fatigue. Visual arts activities require the coordination of fine movements for drawing (Beesley et al., 2011) and rhythmic movement activities involve body coordination and activation (Thornberg et al., 2014). Similar to cognitive training in occupational therapies, visual arts activities can also be cognitively demanding (Beesley et al., 2011). The process of creating artwork needs a certain level of mental energy, and rhythmic movement activities require memorizing several steps or movements (Thornberg et al., 2014). Although the application of art forms was joyful and playful, some stroke survivors still pointed out that the intervention triggered unpleasant or sad memories (Higgins et al., 2005). Therefore, creative arts-based therapies, which are also recognized as a type of psychotherapy, are likely to trigger undesirable thoughts during the process. However, these feelings should be considered as reasonable and a part of the therapeutic process. Nevertheless, the fundamental focus should be placed on how different art modalities can help participants develop insights and skills to cope with their challenges. In some studies, the stroke survivors also commented on feeling confused and unfamiliar with the process at the beginning of the interventions (Beesley et al., 2011; Thornberg et al., 2014), which may have caused some survivors to drop out (Beesley et al., 2011). At the same time, when they persisted until the end, they were able to gain desirable effects from the intervention (Thornberg et al., 2014). It is understandable that not all stroke survivors are able to express themselves through different art forms or be able to articulate the art-making process, perhaps more time for exploration and guidance may be required from the therapists in that respect. It is also recommended that flexibility is allowed in the intervention sessions for the encouragement of diverse participation (Tarrant et al., 2016).

## Strengths and limitations

Although different studies regarding creative arts-based therapies on stroke survivors have been conducted, there remains a lack of common consensus as to which art form can bring about the best results. Different art modalities may benefit different participants in different ways. This qualitative systematic review synthesizes different findings and takes the first step to create an overview on which art media are more useful in achieving certain objectives and experiences in the stroke survivors. The findings suggest that the flexibility of applying different art modalities and the sensitivity of the therapists are vital, and the integrated use of multiple art modalities may also have the special advantage of offering this flexibility.

Nonetheless, there are several limitations to this qualitative systematic review. First, among the included studies, not all the therapies or interventions were led by qualified creative arts therapists. While most music-based interventions were led by qualified music therapists, such as Nordoff-Robbins music therapists and Neurological music therapists, other creative arts-based interventions were led by other professional staff such as nurses and instructors. It should also be noted that it remains unclear as to how the qualification of the interventionists affects participants' perceived experiences in the interventions. Second, this review focuses on the experiences of creative arts-based therapies. As qualitative and mix-method studies were more suited to provide this information, quantitative studies were nevertheless excluded from this review. Third, as nearly half of the studies applied music-based interventions, results from other arts-based interventions may have been under-reported. Fourth, not all themes had corresponding quotations as some studies did not provide quotations for every single theme, especially those that adopted mix-method design. These studies conducted different interviews with the stroke survivors, but the results were not described in as much detail as those in the qualitative studies; hence, the supporting quotations of some specific themes were not reported. Last, the literature search was conducted only on the English written literature. The authors believe that other studies should have been conducted in some areas in which English was not the language used for writing the research reports.

## Further studies

This systematic review included both studies on interventions led by qualified creative arts therapists and by other professionals, the findings are nonetheless not sufficient enough to present the differences in the stroke survivors' perceived experiences between interventions led by qualified interventionists and those led by other professionals. Further studies are required to investigate into this issue, as more and more professionals other than creative arts therapists have started to adopt arts-related interventions to assist the stroke survivors. Apart from that, more empirical findings are needed for creative arts-based therapies, particularly for interventions that adopt multiple art modalities. Further studies may focus on the effectiveness of these interventions on stroke survivors or on other population groups. Other studies may also compare the effectiveness and the participants' experiences between different single art modality interventions and intervention using multiple art modalities.

## Conclusion

Based on the experiences of the stroke survivors, creative arts-based therapies which focus more on psychosocial and spiritual development, demonstrate the potential to supplement existing stroke rehabilitation programs that primarily solely focus on functional recovery. Such comprehensive rehabilitation may provide holistic care and better post-stroke quality of life for the stroke survivors. Different art modalities are perceived to be useful in achieving different therapeutic goals. Interventions that offer opportunities for the participants to experience different art modalities during the process may foster participation and enhance flexibility. Therefore, further research is needed to demonstrate the differential benefits or special advantages in using single or multiple art modalities as well as having qualified therapists in creative arts-based therapies.

## Author contributions

TL conceptualized the review, conducted the searches, data selection, data extraction, and quality assessments. JL crossed-validated the data selection and data extraction and conducted the quality assessments independently. RH revised the manuscript and supervised the entire review. All authors have contributed toward revising the manuscript and have read and approved the submitted version.

### Conflict of interest statement

The authors declare that the research was conducted in the absence of any commercial or financial relationships that could be construed as a potential conflict of interest.
