# Corrigendum: Creative Arts-Based Therapies for Stroke Survivors: A Qualitative Systematic Review

**DOI:** 10.3389/fpsyg.2019.01538

**Published:** 2019-07-02

**Authors:** Temmy Lee Ting Lo, Janet Lok Chun Lee, Rainbow Tin Hung Ho

**Affiliations:** ^1^Department of Social Work and Social Administration, The University of Hong Kong, Hong Kong, Hong Kong; ^2^Centre on Behavioral Health, The University of Hong Kong, Hong Kong, Hong Kong; ^3^Sau Po Centre on Ageing, The University of Hong Kong, Hong Kong, Hong Kong

**Keywords:** creative arts-based therapies, expressive arts therapy, qualitative systematic review, rehabilitation, stroke

In the original article, we neglected to include the funder Research Grants Council of Hong Kong, General Research Fund (GRF/HKU/17609417), to Temmy Lee Ting Lo and Rainbow Tin Hung Ho.

Due to this omission, the following Funding statement will be added to the original article:

This work was supported by the General Research Fund, Research Grants Council of Hong Kong (GRF/HKU/17609417, to TL and RH).

The authors apologize for this error and state that this does not change the scientific conclusions of the article in any way. The original article has been updated.

