# Supplementary material for: Creative Arts-Based Therapies for Stroke Survivors: A Qualitative Systematic Review
Source: Front Psychol. 2018 Sep 25;9:1646. doi: 10.3389/fpsyg.2018.01646 (PMC6158307; doi:10.3389/fpsyg.2018.01646)
Supplement: Supplementary file 1 [file Table_1.pdf]

Table S1. Results of quality assessments

Quality Assessment									
	Credibility					Relevance			Quality
	Q1: Were explanations of sampling strategies and data collection methods provided?	Q2: Was the method of data analysis described and enough data displayed to allow the reader to determine whether the interpretations made by the researcher are supported by the data (auditability)?	Q3: Did the authors acknowledge the influence of the research process and the presence of the researcher including the role of prior biases, assumptions, and experience, on the collected data (reflexivity)?	Q4: Has appropriate attention been given to contradictory data? Are negative cases taken into account?	Q5: Did the authors explore alternative, plausible explanations for the data collected and incorporate a range of different perspectives (fair dealing)?	Q6: Did the authors provide information regarding participants, setting and context so that the reader might be able to determine the relevance of the findings to other settings (transferability)?	Q7: Did the authors discuss findings within a broader context, propose a generalization of findings and/or suggest a direction for future research (analytic generalization)?		
Beesley et al., 2011	✓	✓	✓	✓		✓	✓		Accepted
Fogg-Rogers et al., 2014	✓	✓	✓		✓	✓	✓		Accepted
Guerrero et al., 2014	✓	✓		✓		✓	✓		Accepted
Higgins et al, 2005	✓	✓	✓	✓	✓	✓	✓		Accepted
Morris et al, 2016	✓	✓		✓	✓	✓	✓		Accepted
Sit et al., 2014	✓	✓				✓	✓		Accepted
Street et al, 2017	✓	✓		✓			✓		Accepted
Tamplin et al., 2013	✓	✓	✓			✓	✓		Accepted
Tarrant et al., 2016	✓	✓		✓		✓	✓		Accepted
Thornberg et al., 2013	✓	✓	✓	✓			✓		Accepted
Wolff et al., 2017	✓	✓		✓			✓		Accepted
Dermers et al., 2015	✓			✓					Unaccepted
